# Supplementary figures and images for: Inhibition of LRRK2 kinase activity promotes anterograde axonal transport and presynaptic targeting of α-synuclein
Source: Acta Neuropathol Commun. 2021 Nov 8;9:180. doi: 10.1186/s40478-021-01283-7 (PMC8576889; doi:10.1186/s40478-021-01283-7)

***
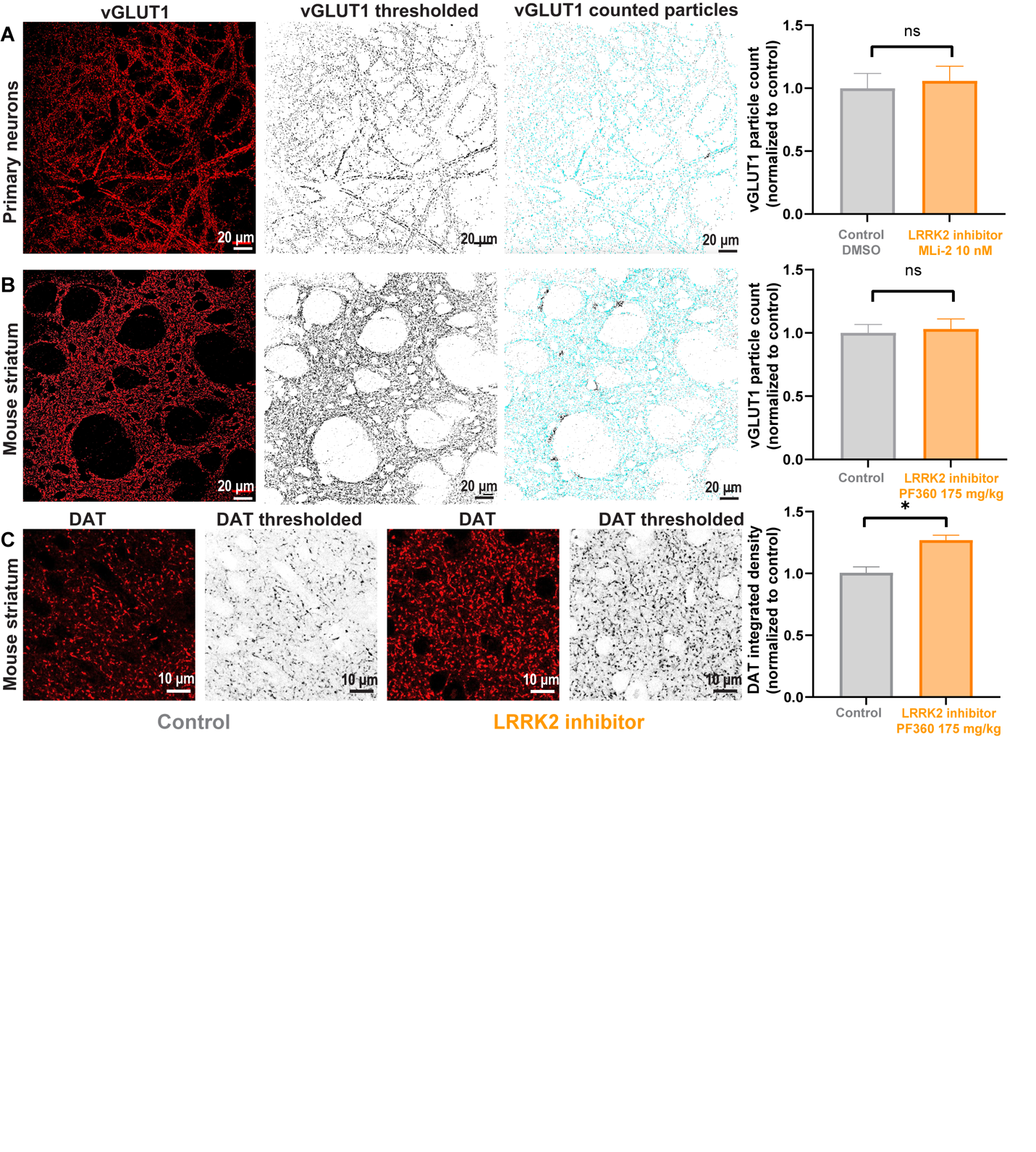
***

Additional file 1: Figure S1

Supplement: Supplementary file 1 — Additional file 1: Fig. S1. No change in glutamatergic terminal density upon LRRK2 kinase inhibitor treatment. A) Shown are confocal images of primary hippocampal neurons stained for vLGUT1 (red), the above-threshold signal (black) and counted particles (bright blue) per frame. The obtained data is quantified in the graph of the right. No change in glutamatergic terminal density was observed for LRRK2 kinase inhibitor treatment. (Nested t-test; t = (58) = 1.16, p = 0.25, n=3, 10 measurements per sample) B) Confocal images of glutamatergic terminals in coronal sections of the mouse striatum, the above-threshold signal (black) and counted particles (bright blue). No change of glutamatergic terminal density was observed upon LRRK2 kinase inhibitor treatment (PF-360). (Nested t-test; t = (58) = 0.88, p = 0.38, n=3, 10 measurements per sample) C) Shows are confocal images of the dorsal mouse striatum stained for DAT (red) and the above threshold signal (black) for control (left) and PF-360 treated animals (right). LRRK2 kinase inhibitor treatment significantly increased the integrated density of DAT signal compared to control, as visualized in the graph on the right. (Nested t-test; t = (4) = 2.99, p = 0.04, *p < 0.05, n = 3, 10 measurements per sample). [file 40478_2021_1283_MOESM1_ESM.docx]

***
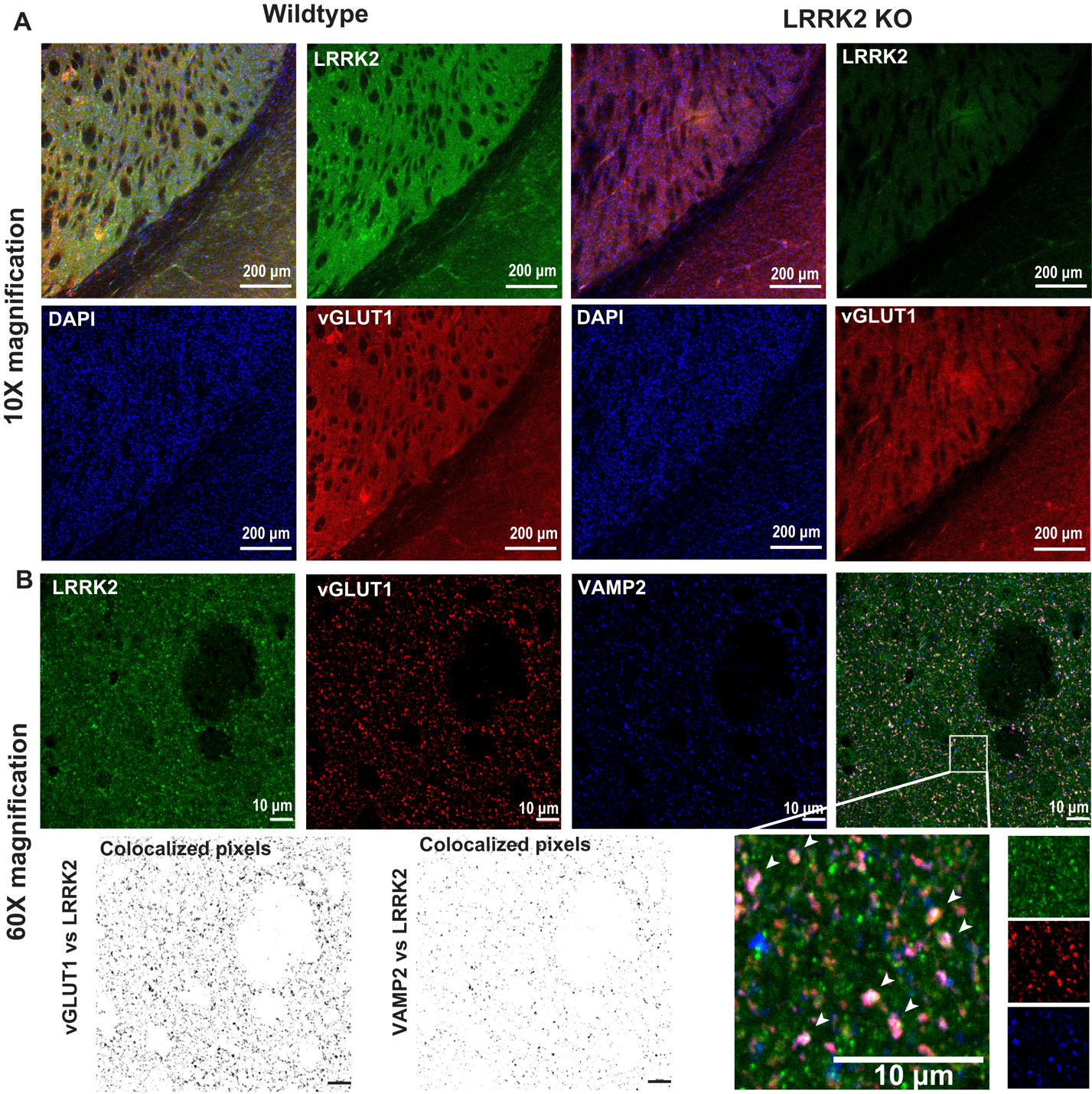
***

Additional file 2: Figure S2

Supplement: Supplementary file 2 — Additional file 2: Fig. S2. Reduction of LRRK2 immunofluorescence signal in LRRK2 KO mice. A) LRRK2 immunofluorescence in C57BL/6J LRRK2 KO mice (kindly provided by Dr. Matthew Goldberg, UAB) is visibly reduced in the striatum and cortex compared to wildtype mice. B) Confocal images of mouse brain sections immunostained for LRRK2 (green) and presynaptic markers (vLGUT1 in red, VAMP2 in blue) show colocalization, especially for glutamatergic terminals in the striatum. [file 40478_2021_1283_MOESM2_ESM.docx]

**
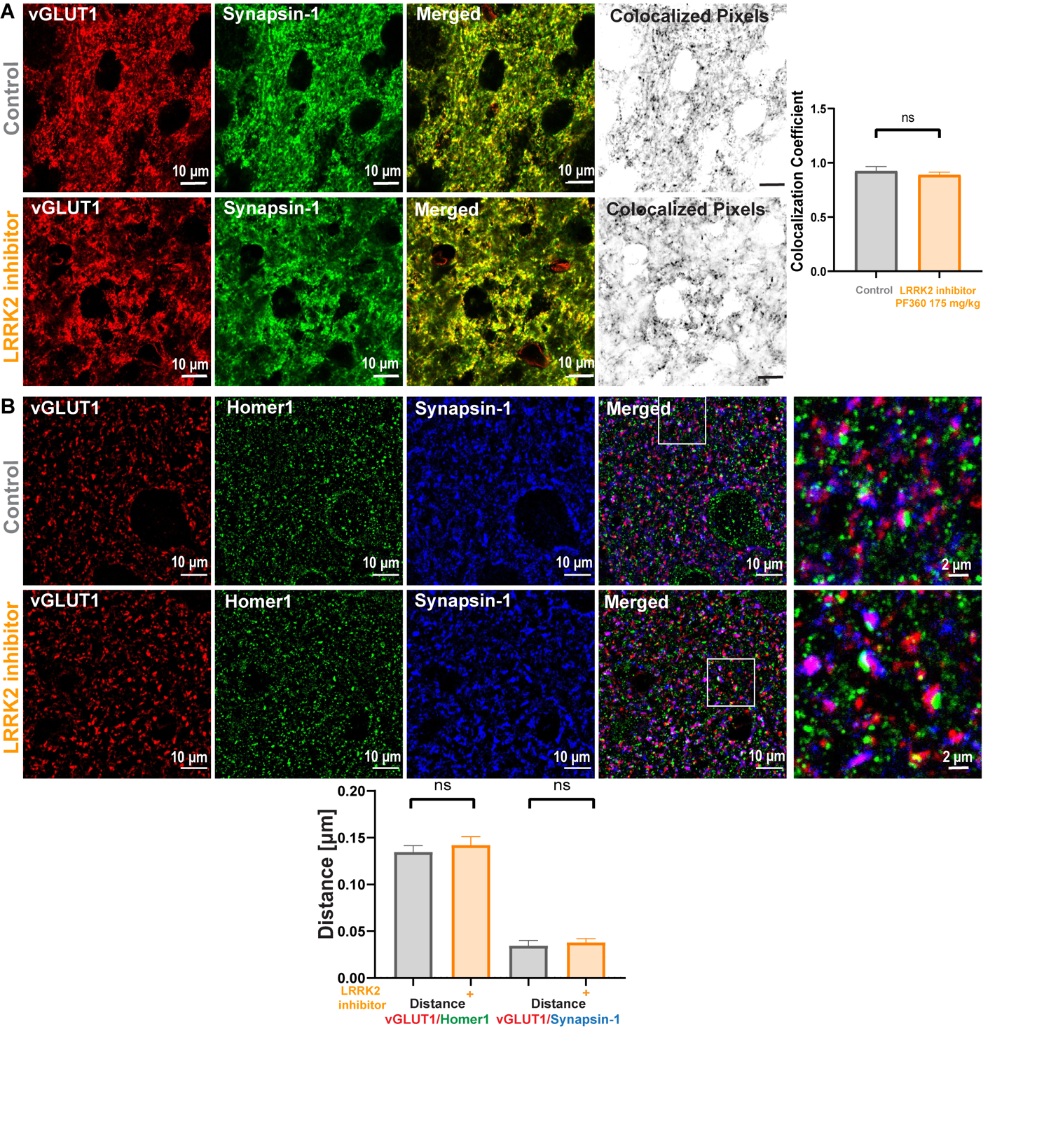
**

Additional file 3: Figure S3

Supplement: Supplementary file 3 — Additional file 3: Fig. S3. No change of localization of synapsin-1 upon LRRK2 kinase inhibitor treatment. A) Colocalization analysis of a mouse cohort treated with LRRK2 kinase inhibitor PF-360 and control mice showed no significant difference in overlap of vGLUT1 (red) and Synapsin-1 (green). Confocal images of the dorsal striatum as well as colocalized pixel maps are shown for visualization. (Nested t-test t = (2) = 0.43. p = 0.71, n = 2, 9 measurements per sample). B) High resolution imaging analysis with ExPath (Expansion Pathology, protocol adapted from Bucur et al. (2020), Nature Protocols) showed no significant change of synapsin-1 (blue) localization at the presynaptic terminal between control animals and PF-360 treated animals. Shown are confocal images of ExPath samples stained for presynaptic markers vGLUT1 (red) and Synapsin-1 (blue) and postsynaptic marker Homer1 (green). (Nested t-test: vGLUT1/Homer1: t = (2) = 0.74, p = 0.54, vGLUT1/synapsin-1: t = (2) = 1.05, p = 0.40, n = 2, 10 measurements per sample). [file 40478_2021_1283_MOESM3_ESM.docx]
